# A revision of *Prespelea* Park (Staphylinidae, Pselaphinae)

**DOI:** 10.3897/zookeys.685.13811

**Published:** 2017-07-20

**Authors:** Michael S. Caterino, Laura M. Vásquez-Vélez

**Affiliations:** 1 Department of Plant & Environmental Sciences, Clemson University, Clemson, SC 29634 USA

**Keywords:** Pselaphinae, Speleobamini, brachyptery, leaf litter

## Abstract

We revise the genus *Prespelea* Park, redefining and redescribing the two previously known species, *P. copelandi* Park and *P. quirsfeldi* Park, and adding ten new species: *P. parki* Caterino & Vásquez-Vélez, **sp. n.**, *P. minima* Caterino & Vásquez-Vélez, **sp. n.**, *P. morsei* Caterino & Vásquez-Vélez, **sp. n.**, *P. divergens* Caterino & Vásquez-Vélez, **sp. n.**, *P. carltoni* Caterino & Vásquez-Vélez, **sp. n.**, *P. myersae* Caterino & Vásquez-Vélez, **sp. n.**, *P. georgiensis* Caterino & Vásquez-Vélez, **sp. n.**, *P. enigma* Caterino & Vásquez-Vélez, **sp. n.**, *P. wagneri* Caterino & Vásquez-Vélez, **sp. n.**, and *P. basalis* Caterino & Vásquez-Vélez, **sp. n.**. The genus is still only known from a relatively small area in the southern Appalachian Mountains, but the diversity is much greater than previously suspected. The new species exhibit considerable diversity in male secondary sexual characters. A preliminary phylogenetic analysis cannot conclusively resolve the polarity of eye and wing reduction across Speleobamini, but the monophyly of Park’s subgenus Fusjugama, if expanded to include all species with full-eyed and winged males, is not supported, and we therefore synonymize it with *Prespelea* *s. str.*

## Introduction

**Figures 1–6.**
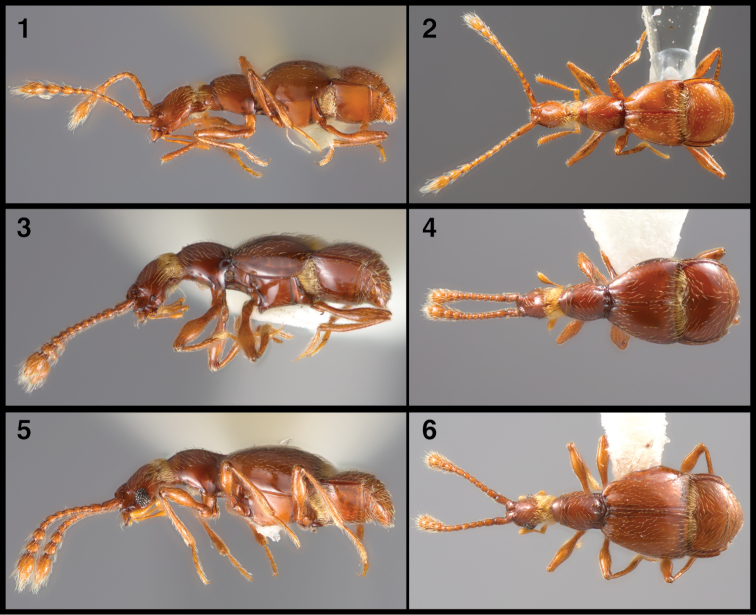
Dorsal and lateral habitus of Speleobamini. **1–2** *Speleobama vana* **3–4** *Prespelea morsei* **5–6** *Prespelea enigma*.

Orlando Park (1953) established the genus *Prespelea* for the new species *P. quirsfeldi*. *Prespelea* and the monotypic *Speleobama* Park (1951) were and remain the only genera within the tribe Speleobamini Park (1951), characterized by an unusual synapomorphy of a deeply dorsally constricted neck (Figure 1) that is largely obscured by dense, opposing fringes of setae. *Speleobama vana* Park, lacking eyes and wings, and having generally elongated body and appendages is an obligate troglobite, known only to occur in McClunney (or McCluney according to some sources) Cave in northern Alabama. *Prespelea*, as the name implies, appeared initially (Park 1953) to represent a less specialized but very similar form, retaining eyes (strongly reduced in *P. quirsfeldi*) but lacking wings, and not exhibiting particularly elongated appendages. When Park (1956) described the second known species, *P. copelandi*, however, the diagnosis of *Prespelea* had to be adjusted considerably, as this species has fully developed eyes and wings (in the male only, as we report here).

To the present day, the tribe Speleobamini has contained only three species, representing an apparent grade from minimally to highly troglophilic. However, no additional work has been done on the group, and the true extent of morphological variability and phylogenetic relationships remain obscure. Through our work and that of others, it has become apparent that this group contains considerably more diversity, which could help illuminate the path into troglophily in this lineage. Here we describe ten additional species, discuss the morphology of the females, and provide a preliminary assessment of phylogenetic relationships in the group.

## Materials and methods

Specimens came from our own collections, and through loans from several institutions:

**CUAC** Clemson University Arthropod Collection, Clemson, SC

**CNCI** The Canadian National Collection of Insects, Ottawa, ON

**FMNH** The Field Museum, Chicaco, IL

**LSAM** Louisiana State Arthropod Museum, Baton Rouge, LA

**UNHC** University of New Hampshire Arthropod Collection, Durham, NH

Morphology was examined using Leica stereomicroscopes, with temporary and permanent slides of selected structures examined using compound microscopes. Males of all morphospecies were dissected in conjunction with attempted DNA extractions (using tissue digestion buffers and proteinase K). For dry mounted specimens the point bearing the specimen was submerged in 100% ethanol for several hours to soften the glue and partially relax the specimen. The specimen was removed from the point and the abdomen was removed by inserting a pin between the metacoxa and 1^st^ visible ventrite. The aedeagus was extracted through the abdominal apex following tissue digestion. Photographs were taken using Visionary Digital’s Passport II imaging system (based on a Canon 6D SLR with 65mm MP-E 1-5× macro lens). Drawings were penciled by hand, traced on a drawing pad, and ‘inked’ in Adobe Illustrator.

Measurements (see Table 1) were taken using a Leica M125 calibrated eyepiece micrometer. Two males and two females of each species were measured, where available. Head length (HL) was measured from the clypeal margin to the upper anterior edge of the neck constriction (ignoring the neck); pronotal length (PnL) was measured along the midline; pronotal width (PnW) was the maximum width, near the midline; elytral length (EL) was measured along the suture from the base of the scutellum to the apex of the suture; elytral width (EW) was the maximum width, invariably near the apices; the 1st visible abdominal tergite length (T3L) was measured along the dorsal midline; total abdomen length (AL) was measured laterally in a straight line from the base of the 1^st^ ventrite to the apex of the last tergite (ignoring telescopy and/or curvature); total length (TL) was calculated) as head length + pronotum length + elytral length + abdomen length.

**Table 1.** Average measurements (in mm) of important body dimensions. N shows numbers of specimens measured for each species.

	**N**	**HL**	**PnL**	**PnW**	**EL**	**EW**	**T3L**	**AL**	**TL**
***P. quirsfeldi***	4	0.37	0.37	0.31	0.51	0.67	0.47	0.62	1.87
***P. parki***	4	0.38	0.36	0.31	0.48	0.68	0.45	0.65	1.86
***P. minima***	2	0.34	0.34	0.31	0.49	0.68	0.46	0.65	1.82
***P. morsei***	4	0.38	0.35	0.32	0.46	0.67	0.46	0.69	1.88
***P. divergens***	3	0.38	0.35	0.31	0.48	0.69	0.46	0.71	1.93
***P. carltoni***	3	0.34	0.33	0.29	0.47	0.63	0.43	0.70	1.84
***P. myersae***	4	0.36	0.34	0.30	0.44	0.61	0.42	0.58	1.73
***P. georgiensis***	4	0.35	0.33	0.30	0.46	0.62	0.41	0.60	1.74
***P. copelandi***	3	0.34	0.33	0.29	0.59	0.67	0.27	0.50	1.75
***P. enigma***	4	0.37	0.33	0.31	0.53	0.65	0.39	0.60	1.83
***P. wagneri***	4	0.36	0.35	0.31	0.54	0.65	0.41	0.55	1.80
***P. basalis***	1	0.39	0.31	0.29	0.59	0.67	0.28	0.51	1.80

All label data were extracted to an Excel spreadsheet, and coordinates were estimated for all localities. This table appears as an Suppl. material 1, while the species treatments provide only brief locality descriptions (aside from the types). Type localities were selected based on availability of DNA sequence data where possible, to reduce ambiguity for future species assignments. In general single-locality type series were preferred, even where male genitalia seemed consistent across localities. A number of unassociated females were recorded from unique localities. These localities are given in the Suppl. material 1.

In order to understand the origins of various characters, particularly the reduction of eyes, flight ability, and general tendency toward a troglobitic morphology, we conducted phylogenetic analyses utilizing both morphological and molecular characters. In addition to previously and newly described *Prespelea* species, we included *Speleobama vana* (for morphology only), and in order to polarize characters within the tribe, further outgroup representatives from the Valdini and Tychini. We scored all taxa for the following morphological characters:

Neck, dorsally: 1. Normal; 2. Deeply cleft and setose.

Neck, ventrally: 1. Flattened beneath, weakly to distinctly carinate laterally; 2. convex beneath.

Male eyes: 1. Well-developed; 2. Poorly developed; 3. Absent.

Male wings: 1. Fully developed; 2. Absent.

Male metaventrite: 1. Unmodified; 2. Produced.

Male metaventral process: 1. N/A; 2. Simple; 3. Apically emarginate to bifid.

Male metatrochanter: 1. Unmodified; 2. Hooked.

Male metatrochanteral process: 1. N/A; 2. Hook basal to medial; 3. Hook apical.

Antennae: 1. Most basal antennomeres no longer than broad; 2. Basal antennomeres slightly longer than broad; 3. Basal antennomeres distinctly longer than broad.

Antennomere 7: 1. Part of gradual sequence; 2. Larger than 6^th^ or 8^th^.

Male 7^th^ ventrite, apex: 1. Shallowly emarginate; 2. Deeply emarginate.

Aedeagus, dorsal plate: 1. Present; 2. Absent.

Aedeagus, shape: 1. Apically narrowed; 2. Hourglass-shaped.

Aedeagus apex, shape: 1. More or less parallel; 2. Expanded.

Aedeagus, apical margin: 1. Apical margin truncate; 2. Apical margin emarginate.

Aedeagus, apicodorsal ridges: 1. Ending short of margin; 2. Extending to margin.

Aedeagus, internal sac: 1. Spineless; 2. With spines.

Female pygidium: 1. Broad, apical margin wide; 2. Smaller, apical margin more distinctly tapered.

Female pygidium: 1. Apically spinose, often with median carina; 2. Not apically spinose or carinate.

Female 7^th^ sternite: 1. With median transverse carina; 2. Without median carina.

Female 7^th^ sternite: 1. Concave in apical half; 2. Convex in apical half.

To help assess variability, relationships, and species limits in the group, we also generated a DNA sequence data set for selected, suitably preserved specimens. We attempted to extract DNA from 22 exemplars representing all 12 species of *Prespelea*, using Thermo Scientific’s GeneJet kit, and amplified 839 bp of the mitochondrial cytochrome oxidase I gene. Outgroup sequences were obtained from our own specimens, and from an unpublished data set in preparation by Dr. Joseph Parker. These included members of the tribe Amauropini (*Arianops*), Valdini (*Valda*), and Tychini (*Custotychus*, *Ouachitychus*, *Tychus*, *Lucifotychus*, and *Nearctitychus*). These were pruned from the base of (invariably monophyletic) Speleobamini for presentation purposes.

DNA sequences were analyzed alone and together with morphological character states using parsimony, and DNA alone was analyzed via maximum likelihood (using a GTR+I+G model with parameter estimates based on one of the most parsimonious trees) as implemented in PAUP* (Swofford, 2002). We experimented with different combinations of outgroups, which did not reveal any effects on ingroup topologies. The full data matrix in nexus format is available as a supplementary file.

## Results

### Phylogeny

We obtained 15 COI sequences representing 8 putatively distinct species. Successful extractions were almost exclusively specimens that had been recently collected directly into ethanol. A few specimens had been previously mounted, but had come directly from 100% ethanol within the past couple years. Sequences have been deposited in GenBank under accession numbers MF380441-MF380455.

**Figures 7–10.**
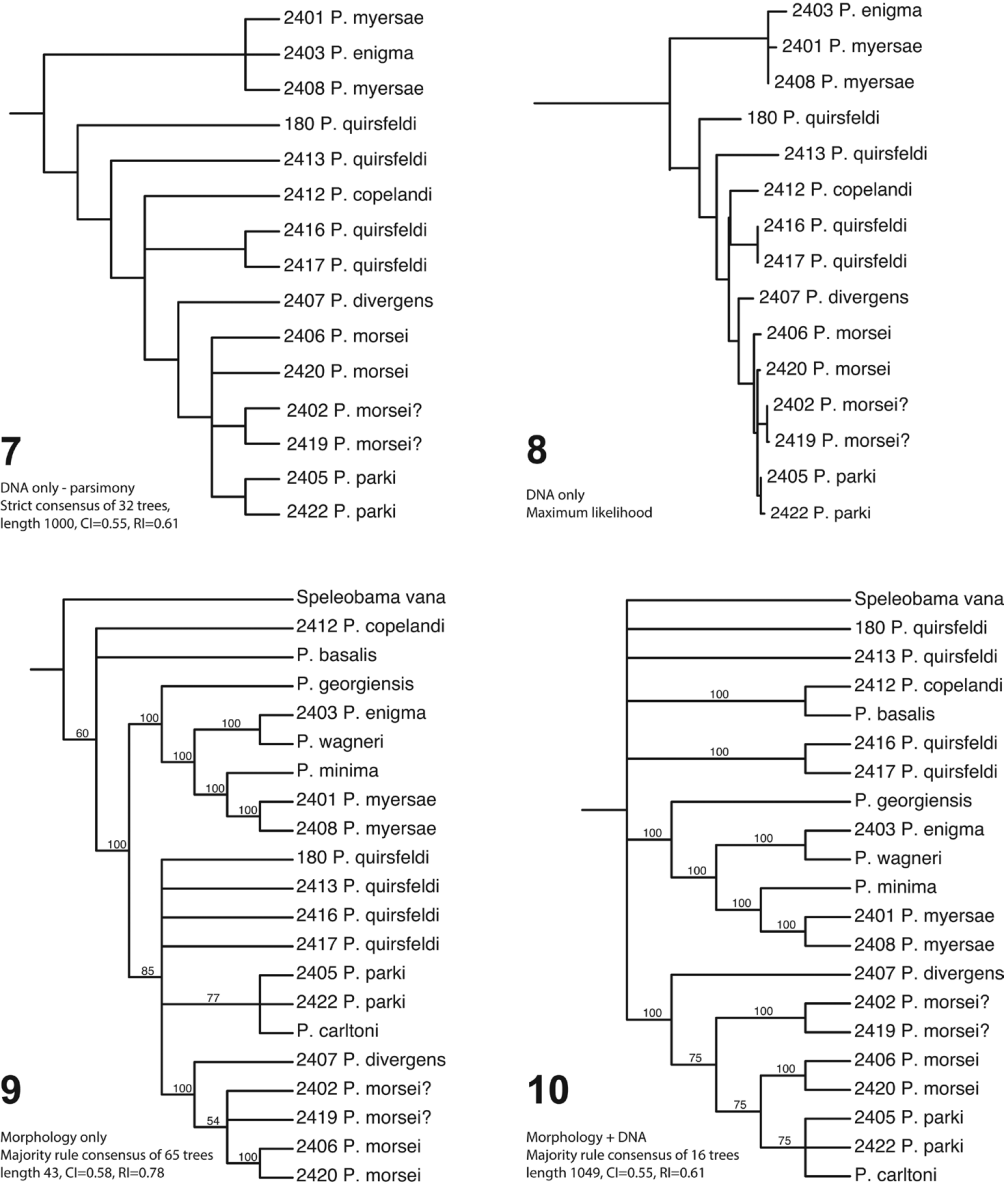
Phylogenetic hypotheses based on: **7** Parsimony analysis of DNA only **8** Maximum likelihood analysis of DNA only **9** Parsimony analysis of morphology only **10** Parsimony analysis of morphology and DNA combined. Numbers on terminal taxa refer to DNA extraction numbers.

Trees based on separate and combined data differ in some details, but agree on some broad, complicated outlines (see Figs 7–10). First, the species with big-eyed males (corresponding to subgenus Fusjugama; i.e. *P. copelandi*, *P. enigma*, *P. wagneri*, and *P. basalis*) do not form a clade in any tree. They are resolved as either a paraphyletic basal grade (morphology; Figs 7, 8) or as a polyphyletic group with various representatives more closely related to either to *P. myersae* (*P. enigma* and *P. wagneri*) or to *P. quirsfeldi* (*P. copelandi* itself). In the combined data (Fig. 10), most big-eyed species group with *P. myersae* (a small-eyed species) and relatives, but not all. This result would make considerable sense in light of the male genitalic morphology, since only some of the ‘*copelandi*’-like species have well-developed internal sac armature like *P. myersae* and *P. minima* do. The aedeagi of other ‘*copelandi*’-like species are little distinguishable from that of *P. quirsfeldi*. However, considering only the more conspicuous eye character states, the result with this male-fully-eyed group basally paraphyletic with respect to reduced eye species makes much more intuitive sense. Nonetheless, the possibility of more vagility in the development of complete eyes is intriguing. Ultimately, better sampling across these groups for sequenceable specimens will be needed to resolve their relationships.

Among the species with reduced eyes in both sexes, all analyses reconstruct *P. quirsfeldi* as a grade subtending a larger group of populations and species. Referring to morphology only, this larger group only includes other reduced-eye species. However, molecular data include one representative of a large-eyed species (*P. copelandi*) within this. Furthermore, uncorrected distances within what we’ve sequenced as *P. quirsfeldi* range to over 6%. Clearly this may suggest that there’s more than one species involved. But we cannot find any morphological differences that would support that possibility. This suggests the possibility that what we are treating as *P. quirsfeldi* may be an old, genetically diverse but morphologically homogenous ancestral stock from which a substantial portion of the genus has arisen. It should further be noted that the *P. quirsfeldi* specimens we’ve sequenced cover a relatively narrow geographical area (as, indeed, the species distribution as a whole does).

The remaining species with reduced-eye males all fall in a well supported clade, most of which are rather minimally divergent in COI (as well as in male genitalia). *Prespelea divergens* falls at the base of this clade in the molecular and combined data trees, consistently 2–3% divergent from the other included taxa. Molecular support for the other morphology-based species (*P. morsei, P. parki*) is considerably weaker, with no divergences exceeding 2%. Although we lack molecular data for *P. carltoni*, morphological data place it within this group as well.

Lacking molecular data, we can say very little about the phylogenetic placement of *Speleobama*. While an assumption of progressive reduction of eyes would suggest its derivation from within *Prespelea* (as is weakly supported by morphological data alone), it is different enough in numerous other characters to cast doubt on this hypothesis. In particular it completely lacks the male secondary characters (metaventrite and metatrochanter) otherwise nearly universal in *Prespelea*. On the other hand, troglobitic habits may be correlated with the absence of distinctive secondary sexual characters in other Pselaphinae (Vásquez-Vélez, unpub. data), for reasons as yet obscure. The male genitalia (as illustrated by Park) are very different from those of any *Prespelea* as well. In the combined data analysis it is equally parsimoniously placed at the base of Speleobamini, what the generic taxonomy would imply, or within a reduced-eye clade, and it is accordingly part of a basal polytomy in the consensus tree.

### Taxonomy

**Tribe Speleobamini Park, 1951: 51**

**Genus *Prespelea* Park, 1953: 251**

#### Fusjugama

Taxon classificationAnimaliaColeopteraStaphylinidae

Park, 1956: 55 (as subgenus) syn. n.

##### Type species.

*Prespelea quirsfeldi* Park (1953: 251), original combination.

##### Diagnosis.

Speleobamini can be easily separated from other North American Pselaphinae by the cervical region of head, which is deeply and narrowly constricted, the constriction obscured by dense fringes of opposing setae. *Prespelea* can be separated from *Speleobama*, the tribe’s only other genus, by the presence of eyes, and by the maxillary palp, in which the fourth palpomere is tuberculate and bearing a long apical ‘cone’; prosternal disk with median setose patch; mesoventrite with well-developed submedian and lateral foveae behind anterior margin; metaventrite with lateral mesocoxal fovea present, small; abdominal ventrite 3 of both sexes with densely setose transverse basal impression; femora obliquely articulated on trochanter so that femur and coxa are relatively close to each other; tarsi of three tarsomeres, the first tarsomere short, the last two very long, the last bearing a single claw; prosternum elongate, without median carina; mesoventrite bisected by strong median carina; procoxae contiguous in confluent cavities; mesocoxae subcontiguous in separate cavities; metacoxae contiguous; males frequently with median metaventral processes and modified metatrochanters; aedeagus large, median lobe elongate, with a long, free style (paramere) on each side that bears four distal setae, and is inserted on the ventral face of the basal capsule.

##### Description.

**Figure 11.**
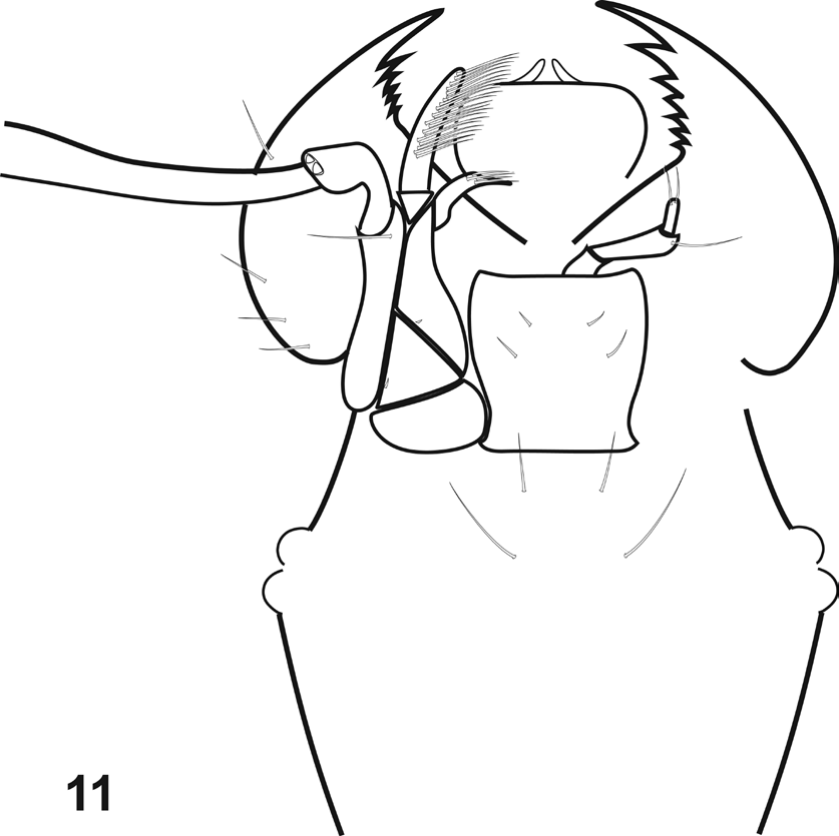
Mouthparts of *Prespelea*, based on *P. myersae*. Left maxilla and right labial palpus are omitted for clarity.

***Size range***: TL 1.54–2.09mm; Max. width (EW) 0.57–0.71mm; **Body.** Integument rufescent, elongate, tapered with prothorax and head narrow; cuticle shining, sparsely setose, most surfaces with moderately long subdecumbent setae, intermixed with longer, finer ‘flying’ setae (these generally appressed in dry specimens). **Head.** HL 0.31–0.41mm; antennal insertions elevated with shallow median depression between them, broadly open laterally and anteriorly; antennae conspicuously setose, with 11 antennomeres: scape cylindrical, about as long as antennomeres 2 and 3 together; antennomere 2 generally about 1.5× length and width of antennomere 3; antennomeres 3–8 generally similar to each other, variable in length among species; antennomeres 9–11 forming weakly distinct club, with length of antennomere 9 about twice that of 8^th^, length of antennomere 10 1.25× that of 9^th^, and apical antennomere about twice as long as 10^th^, with its sides rounded, tapering to subacute apex; eyes present, situated somewhat ventrolaterally, either of 2–4 facets or >30 (no intermediates known); epistoma broad, somewhat produced, finely elevated along apical margin; labrum rounded laterally and apically, subcircular; mandibles (Fig. 11) apically acute, with row of 5–7 serrate denticles along apical half of inner margin; cardo large, weakly projecting, glabrous; stipes triangular, with single small seta near basolateral corner; lacinia short, with few medially directed apical spines; galea long, digitiform, strongly fimbriate on inner margin; maxillary palp with four palpomeres, all appearing smooth and glabrous, with only few inconspicuous setae, the basalmost palpomere short and elbowed, the second the longest, strongly clavate, the third and fourth slightly shorter than second, subequal, more gradually clavate, the fourth bearing an apical digitiform process; submentum indistinct; mentum subquadrate, slightly elongate, with one or two pairs subapical setae; labial palpifer projecting, bearing three palpomeres, the basalmost palpomere very short, second palpomere about half as long as mentum width, weakly expanded apically, apical palpomere thin and short, bearing pair of apical setae. **Thorax.** PnL 0.31–0.37mm, PnW 0.29–0.33mm; pronotum narrow, sides rounded, widest near middle, slightly narrowed to base and apex, with five deep impressions along basal margin, setae of disk converging anteromedially; pronotosternal sutures absent; prosternum with or without vestigial lateral foveae, disk bearing median cluster of setae; prosternal cavities contiguous, broadly open behind; mesoventrite with well-developed submedian and lateral foveae behind anterior margin; metaventrite with lateral mesocoxal fovea present, small; male metaventrite with variably developed process; episterna and epimera concealed. EL 0.39–0.61mm; EW 0.57–0.71mm; elytra strongly narrowed to base (more strongly in wingless forms, including females of all species), each with or without weak pair of basal foveae; sutural stria present; metathoracic wings present (some males) or absent (some males and all females). **Legs.** Femora obliquely articulated on trochanter so that femur and coxa are relatively close to each other; tarsi of three tarsomeres, first short, last two very long, last tarsomere bearing a single claw; males frequently with modified metatrochanters. **Abdomen.** T3L 0.25–0.49; tergite 3 half to two-thirds elytral length (relatively longer in wingless forms), with deep transverse basal impression, densely lined with setae, sides with strong submarginal carina, curving mediad basally; other tergites short, without distinct lateral carinae, only tergites 4 and 5 with distinct paratergites; tergite 7 small and weakly depressed in males, wider and often medially carinate in females; abdominal ventrite 3 of both sexes with densely setose transverse basal impression; ventrites 2 and 3 developed into prominent intercoxal process. **Aedeagus.** Symmetrical, median lobe simple, sides parallel to sinuate to convergent, apex truncate to emarginate, often laterally expanded; apical foramen simple or delimited laterally to subapically by weakly elevated ridges; internal sac simple or bearing spines; parameres elongate, bearing four distal setae, articulated on the ventral face of the basal capsule.

##### Distribution.

The genus is only known from the southern Appalachian Mountains.

##### Remarks.

Little to nothing is known about the natural history of *Prespelea* species. Although their morphology and relationships to true troglobites seem to suggest deep soil or ‘subcave’ preferences, our own group’s recent collections have been from more typical litter samples, principally mixed hardwood litters, frequently under evergreen ericaceous shrubs.

We here synonymize the subgenus Fusjugama Park since the major phylogenetic divisions in the genus do not support the gross large-eye/small eye division on which that name was based.

### Key to species (males only)

**Table:** 

1	Specimens with one or more of the following: fully developed eyes and wings, and/or (if eyes and wings vestigial) with metatrochanters bearing some form of hook; often also with weak to prominent metaventral processes	**2 (Males)**
–	Specimens, if small-eyed, then without modified metaventrite or metatrochanters	**Females (not keyed further)**
2	With fully developed eyes	**3**
–	Eyes reduced to a few ommatidia	**6**
3	Metaventrite unmodified (Fig. 20)	**4**
–	Metaventrite modified (Figs 12–19, 21–22)	**5**
4	Metatrochanteral processes simple tapered hooks near or beyond middle of metatrochanters (Fig. 31)	***P. copelandi* Park**
–	Metatrochanters with rather low, broad hooks near base (Fig. 33)	***P. basalis* sp. n.**
5	Process of metaventrite weakly developed (Fig. 21)	***P. enigma* sp. n.**
–	Process of metaventrite prominent (Fig. 22)	***P. wagneri* sp. n.**
6	Metatrochanters apically extended by hooklike process (Figs 25–27, 29	**7**
–	Metatrochanters with hooklike process medial, not extending from apices (Figs 23, 24, 28, 30)	**10**
7	Metaventral process narrowing to apex (Fig. 14, 15)	**8**
–	Metaventral process basally constricted (Figs 16, 18)	**9**
8	Metaventral process very narrow (Figs 14)	***P. minima* sp. n.**
–	Metaventral process broader (Fig. 15)	***P. morsei* sp. n.**
9	Apices of metaventral process divergent (Fig. 16)	***P. divergens* sp. n.**
–	Apices of metaventral process not divergent, simply divided (Fig. 18)	***P. myersae* sp. n.**
10	Metatrochanteral processes broad and basal (Fig. 28)	***P. carltoni* sp. n.**
–	Metatrochanteral processes narrow and medial to subapical (Figs 23–24, 30)	**11**
11	Metaventral process narrowing to apex (Fig. 19)	***P. georgiensis* sp. n.**
–	Metaventral process broad to apex (Fig. 12–13)	**12**
12	Metaventral process produced anterad (Fig. 13); aedeagus broadened apically (Fig. 36)	***P. parki* sp. n.**
–	Metaventral process not produced anterad, anterior face angled slightly posterad (Fig. 12); aedeagus more or less parallel-sided to apex (Fig. 34)	***P. quirsfeldi* Park**

**Figures 12–22.**
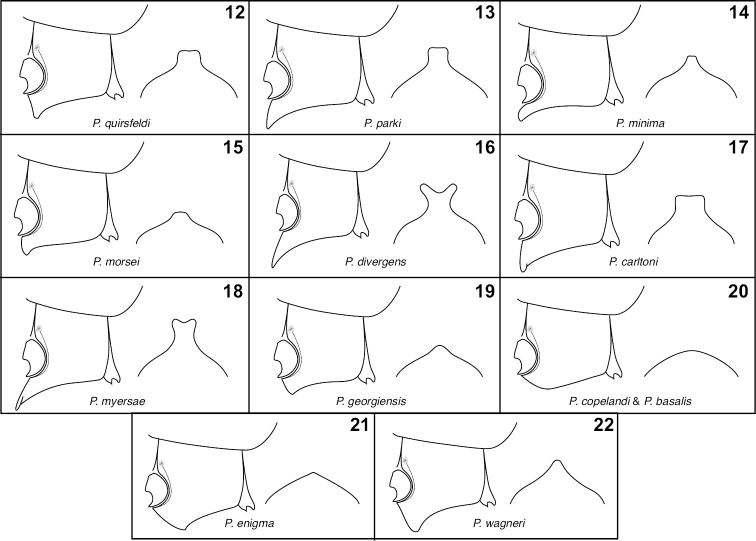
Metaventrites, lateral (left) and posterior (right) views.**12** *P. quirsfeldi* **13** *P. parki* **14** *P. minima* **15** *P. morsei* **16** *P. divergens* **17** *P. carltoni* **18** *P. myersae* **19** *P. georgiensis* **20** *P. copelandi* and *P. basalis* (indistinguishable in this feature) **21** *P. enigma* **22** *P. wagneri*.

**Figures 23–33.**
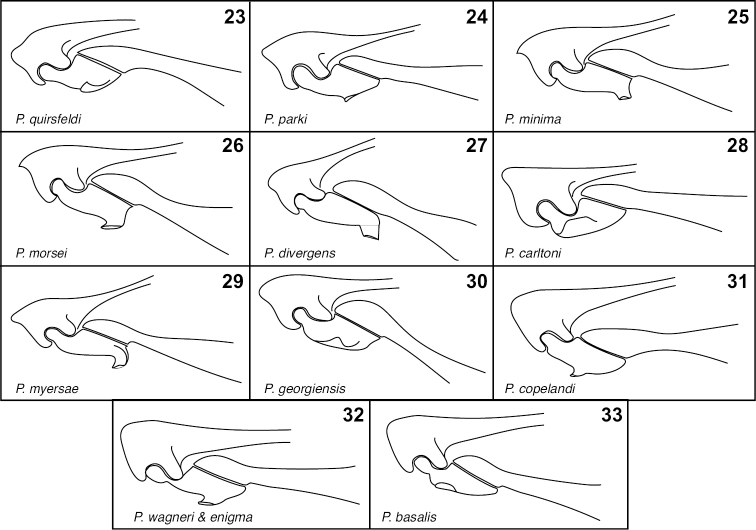
Metatrochanters, ventral view. **23** *P. quirsfeldi* **24** *P. parki* **25** *P. minima* **26** *P. morsei* **27** *P. divergens* **28** *P. carltoni* **29** *P. myersae* **30** *P. georgiensis* **31** *P. copelandi* **32** *P. basalis* and *P. enigma* (indistinguishable in this feature) **33** *P. wagneri*.

#### Prespelea quirsfeldi

Taxon classificationAnimaliaColeopteraStaphylinidae

Park, 1953

**Figures 34–47.**
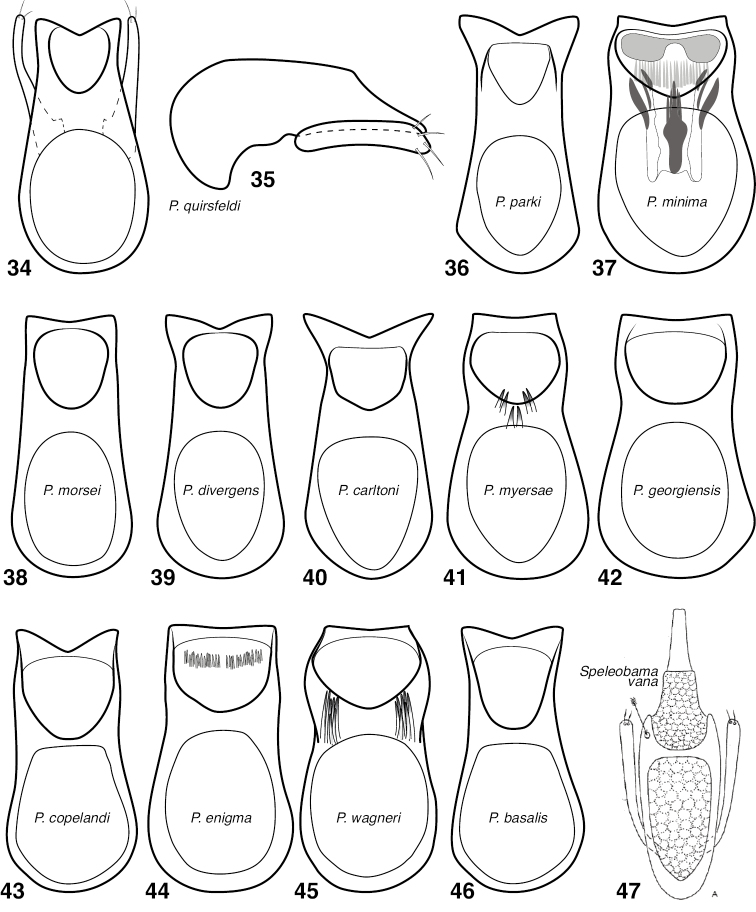
Aedeagus, mostly dorsal view (except 35). Parameres omitted except from **34, 35** and **47**. **34** *P. quirsfeldi* **35** *P. quirsfeldi*, lateral view **36** *P. parki* **37** *P. minima* **38** *P. morsei* **39** *P. divergens* **40** *P. carltoni* **41** *P. myersae* **42** *P. georgiensis* **43** *P. copelandi* **44** *P. enigma* **45** *P. basalis* **46** *P. wagneri* **47** *Speleobama vana* (from Park, 1951).

Figs 12 , 23 , 34–35, Map 48

Prespelea quirsfeldi Park, 1953: 251

##### Type material.

1 paratype male (dissected and slide-mounted by Park) from type locality (“North Carolina, Cataloochee Divide nr. 5000 ft. ele., 12.VI.1940, Quirsfeld leg.”/”Paratype Prespelia [sic] quirsfeldi Park, 4–59” (FMNH). The Holotype male (USNM), collected at the same locality two days later, was not examined. **Other material**: Three paratype females cannot confidently be assigned to species, given the diversity of species occurring in the same general area; for full details see Suppl. material 1.

##### Diagnosis.

Distinguishable only by the following characters of the male: metaventral process rather low, projecting perpendicularly below mesocoxae, apex (in posterior view) broad, subtruncate to very weakly emarginate; metatrochanter with hook subapical, with a moderately broad base tapering to subacute tip; aedeagus with sides convergent from basal third, weakly widening to apex, apex distinctly emarginate, apicodorsal ridges ending short of distal corners; internal sac lacking spines. Female pygidium with weak median process; apical ventrite slightly bilobed. TL 1.83–1.90mm; Max. width (EW) 0.67–0.69mm.

##### Distribution.

Known from three somewhat disjunct localities, Cataloochee Divide and Cades Cove within Great Smoky Mountains National Park, and around the Coweeta Hydrological Laboratory south of Franklin, NC.

##### Remarks.

This species was described from five specimens, two males and 3 females. The types were collected from ‘deep leaf mold in thickets of rhododendrons’ (Park, 1953). ‘Leaf mold’ and ‘rhododendron duff’ have been mentioned on subsequent specimen labels as well, as has ‘nr. rotten wood’. Given the diversity of *Prespelea* now evident, and the difficulty to impossibility of associating females, the paratype females must be considered only tentatively conspecific.

There are unfortunately few subsequently collected specimens that we can definitely attribute to this species. It appears to be somewhat widely distributed, ranging from localities near the type locality along the Cataloochee Divide (Great Smoky Mountains National Park – GSMNP) 60 km west to Cades Cove (also GSMNP) and over 60 km south to the Coweeta area, and it exhibits some variation in metaventral process shape, metatrochanteral hook shape, and even in aedeagal shape. Furthermore, externally many specimens appear almost indistinguishable from those of *P. parki*, which is distinct based on both aedeagal morphology and available sequence data. Therefore there are a number of specimens that we have identified only as ‘*P. quirsfeldi* or *parki*’. The deep genetic diversity further underscores the need to do further work in this complex to resolve species limits and relationships.

Some of the specimens we cite as ‘other material’ were initially labeled by John Wagner as types of his manuscript species ‘*P. steevesi*’ and ‘*P. coweeta*’. We do not believe these constitute distinct species, but have left his labels on the specimens.

#### Prespelea parki

Taxon classificationAnimaliaColeopteraStaphylinidae

Caterino & Vásquez-Vélez sp. n.

http://zoobank.org/E211E5FD-6DC6-42A6-852C-D6BB81D2C502

Figs 13 , 24 , 36, Map 48

##### Type material.

**Holotype male**: NC: Graham County, Joyce Kilmer Memorial Forest, near junction of Indian and Santeetlah Creeks, 35.3451°N, 83.9670°W, vi.24.2015, S. Myers & M. Caterino, sifted litter, CUAC000010972 (DNA extract MSC-2405); deposited in FMNH. **Paratypes (2)**: male (CUAC000010948) and female (CUAC000010964; DNA extract MSC-2422) with identical data to type. **Other material**: Macon Co., NC and Union Co., GA; for full details see Suppl. material 1.

##### Diagnosis.

Distinguishable from *P. quirsfeldi* only by the following characters of the male: metaventral process more laminate, and slightly more projecting anterad, apically weakly emarginate; metatrochanter with laminate subapical tooth, very similar to that of *P. quirsfeldi* (identical in some, but broader and more flangelike in others, particularly Kilmer specimens); mesofemora somewhat swollen. Aedeagus with sides converging from basal third to near apex, weakly sinuate then strongly divergent to weakly rounded apical corners, apical margin strongly emarginate; apicodorsal ridges strong, converging toward apex, ending freely (apicodorsal foramen only weakly closed). Female pygidium with median carina increasing to apex, apical ventrite weakly bilobed; neck convex beneath, with distinct median ridge and cluster of postgular setae. TL 1.82–1.91mm; Max. width (EW) 0.66–0.71mm.

##### Distribution.

Southwestern North Carolina, extending southwestward to Brasstown Bald in northeastern GA.

##### Remarks.

As discussed above, there is a relatively broad range of variation between *P. quirsfeldi* and what we name as *P. parki*, with some specimens falling between. Thus, outside of type material from the Joyce Kilmer Memorial Forest, which we have been able both dissect and sequence, and which is distinct in both morphological and molecular characters, specimens from other localities listed above are merely ‘affiliated’ with one or the other species. A number of other specimens from localities in and around Great Smoky Mountains National Park cannot be confidently attributed to either (despite dissection). See Suppl. material 1 for additional possible localities.

We name this species for Orlando Park (1901–1969), a leading 20^th^ century specialist in Pselaphinae, and author of the genus. One of the specimens we cite as ‘other material’ was initially labeled by John Wagner as a ‘type’ of his manuscript species ‘*P. parki*’. While we have used his intended name, but have left his ‘labels on the specimen, we exclude this from our type series.

#### Prespelea minima

Taxon classificationAnimaliaColeopteraStaphylinidae

Caterino & Vásquez-Vélez sp. n.

http://zoobank.org/CC3AC822-AB1B-4053-898B-A457A5C46035

Figs 14 , 25 , 37, Map 49

##### Type material.

**Holotype male**: TN: Sevier Co., Great Smoky Mountains National Park, Beech Gap on Clingman Dome Rd. at Appalachian Trail crossing [35.61°N, 83.45°W], 1750m, VI.28.2001, forest litter, C. Carlton, A.K. Tishechkin, & V. Moseley (LSAM0096333); deposited in FMNH. **Paratypes (2)**: 1 male: same data as type; 1 male: GSMNP: Chimneys Picnic area (DNA extract MSC-2415); for full details see Suppl. material 1.

##### Diagnosis.

Distinguishable only by the following characters of the male: metaventral process rather small, narrowing to subtruncate or weakly emarginate apex (in posterior view), distinctly projecting anterad between mesocoxae; metatrochanters with hooks extending apically, laminate, moderately broad, with truncate apex. Antennae slightly elongate; neck flattened beneath, subcarinate ventrolaterally. Aedeagus broad, sides sinuate, apex emarginate, apicodorsal ridges curving inward at apex; apical foramen with lightly sclerotized plate across apex; internal sac with strong medial and lateral spines, ventrally with ~20 minute spines. TL 1.80–1.84mm; Max. width (EW) 0.67–0.69mm.

##### Distribution.

Known only from two localities in the central part of GSMNP.

##### Remarks.

Samples were noted to have been taken only from ‘forest litter’. Despite an attempted DNA extraction from an older mounted specimen, we have not been successful in generating a DNA sequence for this species.

The species is named for its small metaventral process.

#### Prespelea morsei

Taxon classificationAnimaliaColeopteraStaphylinidae

Caterino & Vásquez-Vélez sp. n.

http://zoobank.org/C7C58731-D799-4C4D-8C34-A2C4753183F2

Figs 3–4 , 15 , 26 , 38, Map 49

##### Type material.

**Holotype male**: NC: Macon Co., Balsam Mountain Preserve, nr. Sugarloaf Creek, 35.3707°N, 83.1108°W, VI.20.2015, S. Myers, sifted acidic cove litter (CUAC000026234; DNA extract MSC-2406); deposited in FMNH. **Paratypes (13)**: several localities within Balsam Mountain Preserve, from oak and mixed oak-hickory litters, all in June 2015; see Suppl. material 1 for details. We also assign two specimens from McDowell Co., NC to this species as nontypes, with some reservation (see remarks).

**Figure 48.**
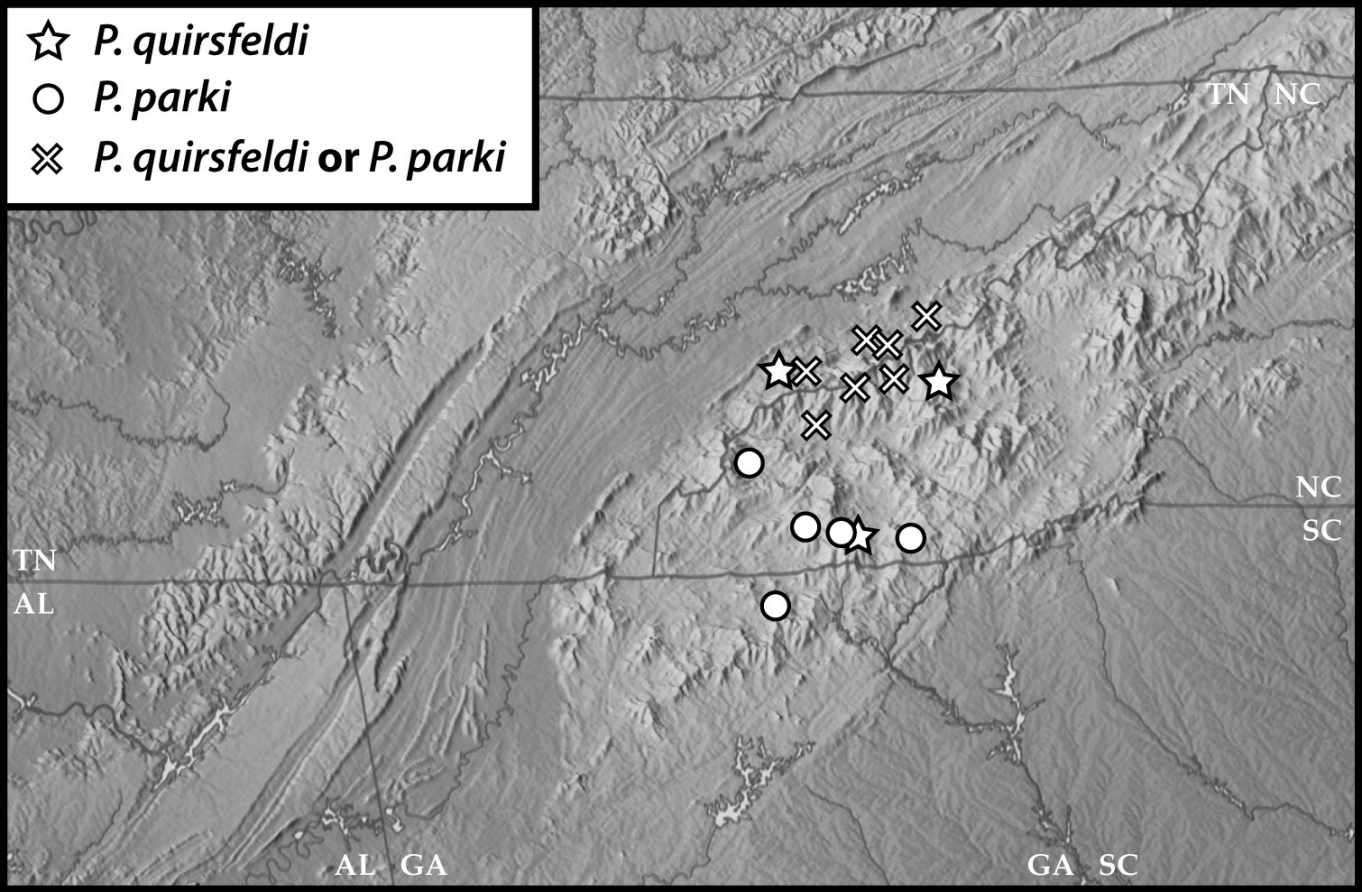
Map of southern Appalachia with specimen records for *P. quirsfeldi*, *P. parki*, and for a number of specimens which we cannot positively identify as one or the other.

##### Diagnosis.

Distinguishable only by the following characters of the male: metaventral process weaker than in *P. quirsfeldi*, but similar; metatrochanteral hook forming moderately broad flange from apex of trochanter; antennomeres subquadrate, basal antennomeres about as long as wide; aedeagus with sides convergent to near apex, apical margin very weakly emarginate; apicodorsal ridges divergent to apical corners, apicodorsal foramen open. Female pygidium essentially unmodified, with very weak median elevation, almost imperceptible until apex; apical ventrite very weakly bilobed; neck convex beneath, with distinct median ridge (not carina) and cluster of postgular setae. TL 1.74–2.09mm; Max. width (EW) 0.65–0.69mm.

##### Distribution.

This species is known only from a relatively small area within the Balsam Mountains of western North Carolina.

##### Remarks.

This species is closely related to *P. divergens* and *P. parki*. The two specimens we attribute to this species from Courthouse Falls, in the Pisgah National Forest of McDowell Co., NC, are particularly vexing. These have identical male genitalia to *P. divergens*, but a more moderate metaventral process like *P. morsei*. The male metatrochanter is also more like that of *P. morsei*, lacking the extreme apical point of the *P. divergens*. Molecular data separate these slightly from either species, but place them considerably closer to *P. morsei*.

This species is named to honor Dr. John Morse, the senior author’s predecessor as director of the Clemson University Arthropod Collection. All specimens of this species were collected in the vicinity of a property owned by John and his wife Suzanne, and their hospitality and assistance were invaluable in carrying out the work.

#### Prespelea divergens

Taxon classificationAnimaliaColeopteraStaphylinidae

Caterino & Vásquez-Vélez sp. n.

http://zoobank.org/CE171B75-3141-4860-AFF1-DB83094AD85A

Figs 16 , 27 , 39, Map 49

##### Type material.

**Holotype male**: SC: Pickens Co., Sassafras Mt., 35.0634°N, 82.7760°W, S. Myers, vi.10.2015, sifted leaf litter (CUAC000025607); deposited in FMNH. **Paratype (1)**: male: same general locality and date as type, but at 35.0579°N, 82.7705°W (CUAC000025636; DNA extract MSC-2407). **Other material**: Two specimens from Macon Co., NC also appear to correspond to this species; for full details see Suppl. material 1.

**Figure 49.**
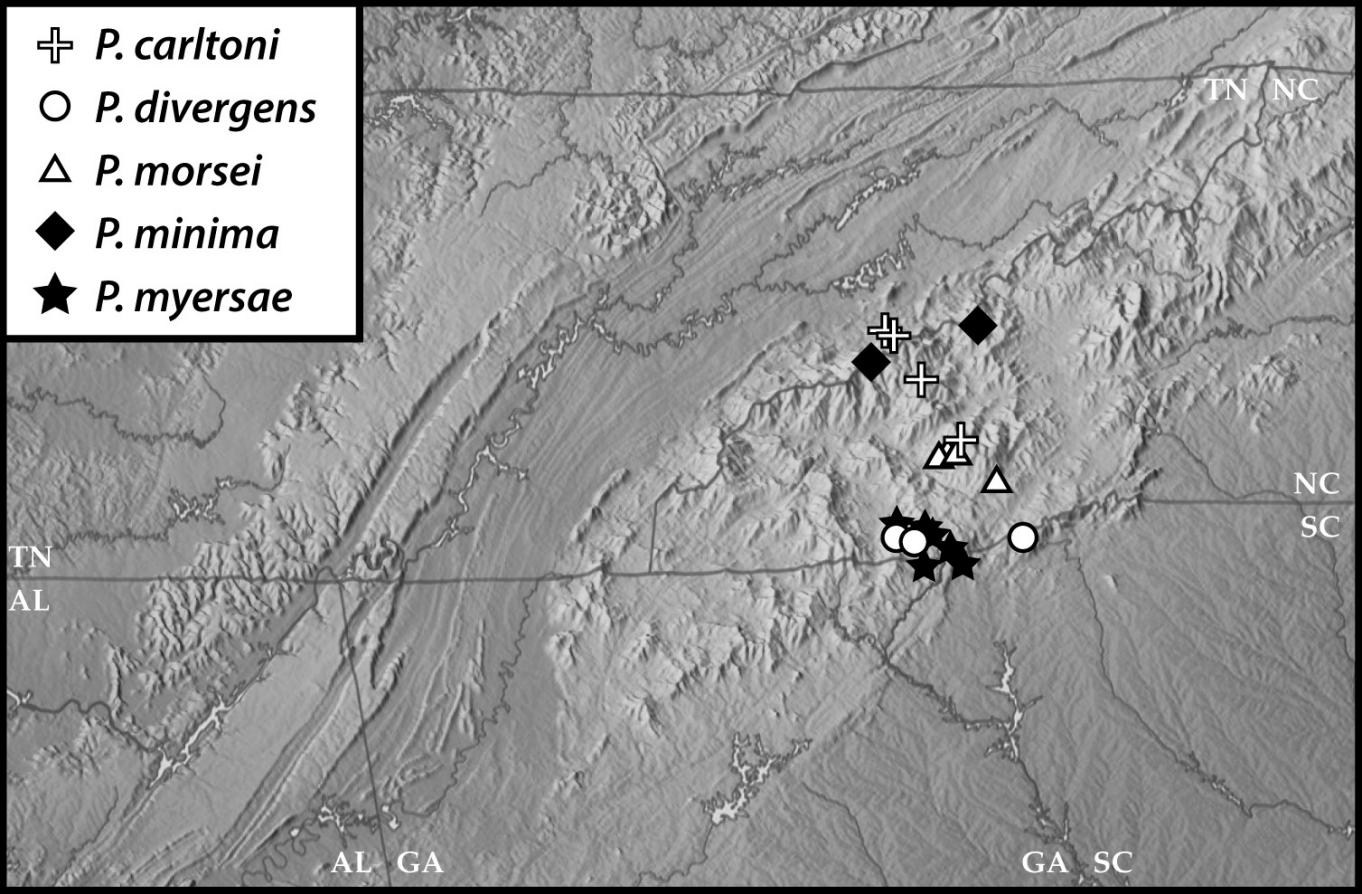
Map of southern Appalachia with specimen records for *P. carltoni*, *P. divergens*, *P. morsei*, *P. minima*, and *P. myersae*.

##### Diagnosis.

Distinguishable from *P. quirsfeldi* only by the following characters of the male: metaventral process strongly projecting anterad, sublaminate, apically divergent; metatrochanter apically extended, with broad recurved flange whose apical corner is strongly produced and acute; antennae weakly elongate; aedeagus with sides convergent to near apex, then weakly divergent to apical corners, apical margin strongly emarginate; apicodorsal ridges divergent to near apices, apical foramen weakly closed. TL 1.83–2.04mm; Max. width (EW) 0.69–0.71mm.

##### Distribution.

This species is only definitely known from Sassafras Mt., South Carolina, the highest point in the state. The other possible locality near Highlands, NC lies about 30 km west.

##### Remarks.

This species’ morphological distinctness is supported by reasonably clear genetic divergence, at least at the one locality and specimen for which we have sequence data.

#### Prespelea carltoni

Taxon classificationAnimaliaColeopteraStaphylinidae

Caterino & Vásquez-Vélez sp. n.

http://zoobank.org/89C5ADC1-4DB3-4BD8-B645-A18F5E888754

Figs 17 , 28 , 40, Map 49

##### Type material.

**Holotype male**: NC: Haywood Co., GSMNP, Cataloochee Rough Ridge Tr., (lower), 306360E, 3940881N [35.5927°N, 83.1374°W], C. Carlton, 7/29/2002, moist berlese (LSAM0092266; DNA Extract MSC-2411); deposited in FMNH. **Paratypes (3)**: 1 male same data as type (LSAM0092265); 1 male: NC: Haywood Co., GSMNP, Cataloochee Rough Ridge Tr., (upper), 305891E, 39040519N [35.5894°N, 83.1415°W], C. Carlton, 7/29/2002, moist berlese (LSAM0060036); 1 male: NC: Jackson Co., Blue Ridge Parkway, nr. Grassy Ridge Mine [35.41°N, 83.05°W], 1520m, A. Smetana (CNC Coleoptera Barcode Voucher 00162876). **Other material**: Two other males (not dissected) from GSMNP, in Cocke Co., TN; for full details see Suppl. material 1.

##### Diagnosis.

Distinguishable from *P. quirsfeldi* only by the following characters of the male: metaventral process, broad, elevated, somewhat blunt at middle, with acute distal margins limited to lateral corners, slightly concave behind; metatrochanter with hook broad and basal. Antennae varied, antennomeres 9 and 10 distinctly transverse in some individuals, more equilateral in others. Tegmen tapering from near base to near apex, abruptly widened and bifurcate at apex, with apical corners subacute; apicodorsal ridges moderate, apical foramen broadly open; internal sac without distinct sclerotizations. TL 1.76–1.96mm; Max. width (EW) 0.45–0.49mm.

##### Distribution.

This species is only known from a few localities within Great Smoky Mountains National Park.

##### Remarks.

This species is most distinctive in its male metatrochanteral process, which is basal and broad. It shows some minor variability in the shape of the metaventral process, which may be weakly emarginate apically or not. Despite an attempted DNA extraction from an older mounted specimen, we have not been successful in generating a sequence for this species.

We name this species for Dr. Chris Carlton of the Louisiana State Arthropod Museum, who collected the types, and who has led efforts to document the beetle fauna of the Smoky Mountains.

#### Prespelea myersae

Taxon classificationAnimaliaColeopteraStaphylinidae

Caterino & Vásquez-Vélez sp. n.

http://zoobank.org/C58B41FE-7FF1-41BD-8DD3-CC1571A2802F

Figs 11 , 18 , 29 , 41, Map 49

##### Type material.

**Holotype male**: “USA: SC: Oconee Co., 34.9899°N, 83.0724°W, Indian Camp Ck, V.04.2015, M.Caterino & S. Myers, Sifted leaf litter” (CUAC000010576); deposited in FMNH. **Paratypes (8)**: 1 male: “USA:SC: Oconee Co., 34.9886°N, 83.0729°W, Indian Camp Ck, V.04.2015, M.Caterino & S. Myers, Sifted leaf litter” (CUAC000010698, DNA Extract MSC-2408); deposited in CUAC. 2 males and 1 female: “USA:SC: Oconee Co., 34.9903°N, 83.0723°W, Indian Camp Ck, V.04.2015, M.Caterino & S. Myers, Sifted leaf litter” (CUAC000010645, CUAC000010631, CUAC000010647); deposited in FMNH, LSAM & CUAC. 1 female: “USA:SC: Oconee Co., 34.9846°N, 83.1018°W, East Fork, V.04.2015, M.Caterino & S. Myers, Sifted leaf litter” (CUAC000010746); deposited in CUAC. 2 females: “USA:NC: Macon Co., 35.0096°N, 83.1245°W, Ellicott Rock Trail, VII.18.2015, S. Myers, Sifted litter” (CUAC000011201, DNA Extract MSC-2421; CUAC000011216); deposited in CUAC. **Other material**: 13 specimens from Macon & Jackson Cos., NC, and Rabun Co., GA; for full details see Suppl. material 1.

##### Diagnosis.

Distinguishable from *P. quirsfeldi* only by the following characters of the male: metaventral process more distinctly laminate and anteriorly projecting than in *P. parki*, weakly to strongly apically cleft; metatrochanter apically produced, with strong, scooplike apical hook; mesofemora somwhat swollen; aedeagus with sides convergent to near apex, then weakly divergent to apical corners; apicodorsal ridges strong, divergent subapically, converging short of apex to weakly closed apical foramen, apical margin subtruncate to weakly emarginate; internal sac with six distinct teeth. Female pygidium weakly depressed, apical ventrite with median transverse carina strongly bilobed, elevated, defining posterior face coplanar with pygidium; neck ventrally flattened, but without median or lateral carinae. TL 1.54–1.84mm; Max. width (EW) 0.57–0.63mm.

##### Distribution.

This species is known from a limited area around the point where North Carolina, South Carolina and Georgia meet, centered on the Ellicott Rock Wilderness.

##### Remarks.

There is slight variability in the form of the metatrochanteral process in specimens from around Highlands, North Carolina, where its apex is slightly widened and truncate. We have dissected one male from this locality and find its aedeagus to be basically similar in overall shape to that of the type, as well as in the distinctive teeth of the internal sac. There is also considerable variation in the widening and emargination of the metaventral process, even among specimens from near the immediate type locality. The female pygidium of this species shows commonalities with the ‘copelandi-like’ (fully-eyed) species of the genus, suggesting that the reduction of male eyes may be quite labile. In addition to several ‘leaf litter’ labels, some specifically mention rhododendron and hemlock as important elements of the sampled microhabitats.

The species is named to recognize the contributions of former Caterino lab postdoc Dr. Shelley Myers, collector of many of the specimens of this and others of the new species described in this paper. Several specimens from the John Wagner collection (FMNH) bear ‘type’ and ‘paratype’ labels, and the manuscript name *P. suteri*. We have left these labels on the specimens, though we do not recognize these as types and the name ‘*P. suteri*’ has no formal status.

#### Prespelea georgiensis

Taxon classificationAnimaliaColeopteraStaphylinidae

Caterino & Vásquez-Vélez sp. n.

http://zoobank.org/A7A7E1D6-5EE8-4B0D-9D9E-332640FC0B66

Figs 19 , 30 , 42, Map 50

##### Type material.

**Holotype male**: “Cloudland Canyon S.Pk., Dade Co., GA. 7.VII.62, forest floor debris” / “H.R. Steeves Jr. Collection” / “CHNM 1963, H.R. Steeves Jr. Pselaphidae Colln. Acc. Z-13, 288”; deposited in FMNH. **Paratypes (7)**: 2 males: same data as type; FMNH. 3 males & 1 female: same locality as type, but collected on ix.3.1961 in ‘debris nr. log’, by W. Suter & J. Wagner; FMNH & CUAC. 1 female: same locality, but collected on ix.1.1961; FMNH. 1 female: Cloudland Canyon State Park, 34.8152°N, 85.4850°W, ix.17.2006, by Igor Sokolov; LSAM0108983. **Other material**: 1 female: TN: Bledsoe Co., Fall Creek Falls St. Park, ix.9.1961, J. Wagner & W. Suter; for full details see Suppl. material 1.

**Figure 50.**
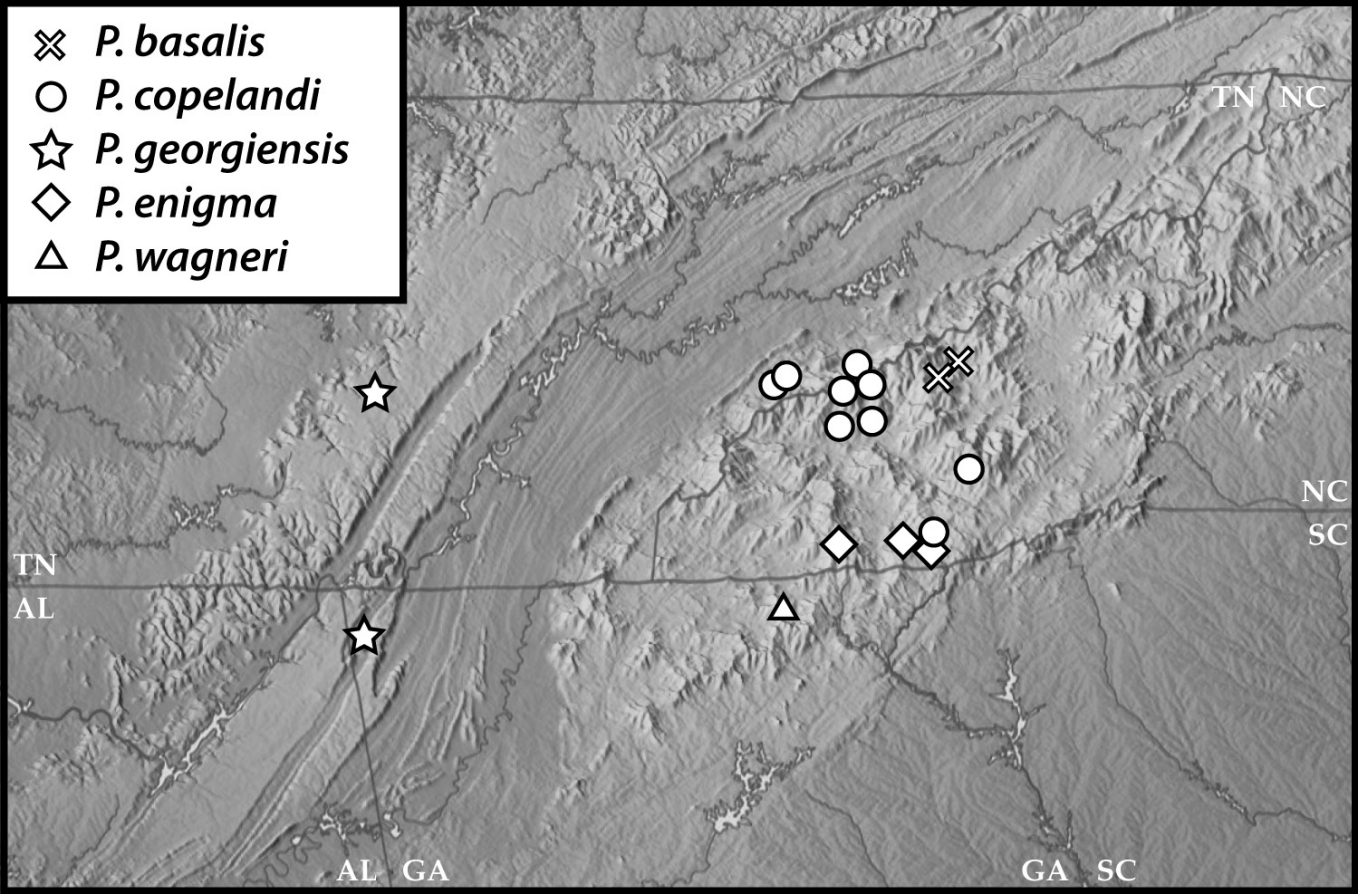
Map of southern Appalachia with specimen records for *P. basalis*, *P. copelandi*, *P. georgensis*, *P. enigma*, and *P. wagneri*.

##### Diagnosis.

Distinguishable from *P. quirsfeldi* only by the following characters of the male: metaventral process forming low, single, blunt medan point; metatrochanteral point short and medial to subbasal, shorter and more basal than that of *P. quirsfeldi*; antennae relatively short, antennomeres 9 and 10 distinctly wider than long; aedeagus with sides sinuate, widened subapically, then weakly convergent to apical corners, apical margin very shallowly emarginate; apicodorsal ridges weak, converging, weakly closing apical foramen; internal sac lacking teeth. Female pygidium flat, moderately broad, rounded apically; apical ventrite with weak transverse median ridge; neck flattened beneath, subcarinate laterally. TL 1.69–1.81mm; Max. width (EW) 0.59–0.65mm.

##### Distribution.

In addition to the type locality, Cloudland Canyon State Park in northwest Georgia, this species may occur about 75 km N in Bledsoe County, Tennessee, but this record is based on a single female, and should be confirmed with more material.

##### Remarks.

Despite its small-eyed males, this species shares a number of characteristics with the large-eyed species related to *P. copelandi*. The male metatrochanter is particularly similar to that of *P. enigma*, as is the metaventral process. The flat and broad female pygidium also allies it more closely with *P. copelandi* than with most of the preceding species (with the possible exception of *P. myersae*). Morphological phylogenetic analyses support this assertion, but it would be good to confirm with molecular data. An attempted DNA extraction from a paratype specimen failed to produce amplifiable DNA.

Some specimens of this species from the John Wagner collection (FMNH) bear ‘type’ and ‘paratype’ labels, and we have used his manuscript name for this species. However, though we’ve left these labels on the specimens, we have selected a different specimen for our primary type than he intended.

#### Prespelea copelandi

Taxon classificationAnimaliaColeopteraStaphylinidae

Park, 1956

Figs 20 , 31 , 43, Map 50

Prespelea copelandi Park 1956: 55

##### Type material.

**Holotype male**: “Cades Cove, Blount Co. Tenn. Berlesed, C.D. Copeland”/ “Type”/ “Field Mus. Nat. Hist. Orlando Park Pselaphidae Colln.” (FMNH). **Other material**: known from nine non-type specimens from Blount and Sevier Cos., TN, and Swain Co., NC (all within Great Smoky Mountains National Park); and Jackson and Macon Cos., NC, east of GSMNP; for full details see Suppl. material 1.

##### Diagnosis.

*Prespelea copelandi* is unique in the genus in lacking male metaventral modifications. The male’s metaventrite is slightly more convex than that of the female, but lacks any distinct process. Like several new species, the males exhibit well-developed eyes and wings (associated females of these species all have reduced eyes with 2–4 ommatidia and undeveloped flight wings); metatrochanter with narrow, acute tooth borne slightly basad midpoint; neck moderately flattened beneath, with median ventral carina; aedeagus with weakly sinuate sides and a deeply emarginate apex. Females: none definitely associated. TL 1.72–1.80mm; Max. width (EW) 0.65–0.69mm.

##### Distribution.

Known from scattered localities within Great Smoky Mountains National Park, as well as a few locations further east and southeast.

##### Remarks.

This species was described from a single undissected male, without associated females. No illustrations were provided. However, the lack of a distinct metaventral process distinguishes it from the other fully-eyed and winged (in males) species we describe below. We assign a few specimens here that do exhibit an extremely minute metaventral denticle, which places them somewhere between this and the next species, and it is this form whose genitalia is illustrated in Fig. 43; we did not risk dissecting the unique type. There is substantial variation, even in male genitalia, with some specimens approaching the shape of *P. quirsfeldi*, with the aedeagus distinctly and evenly narrowed subapically. There is also variation in the depth of the apical emargination of the aedeagus, and this species thus remains poorly characterized. Further material from the type locality (Cades Cove) that can be dissected and sequenced would help define what should and shouldn’t be assigned to *P. copelandi*.

#### Prespelea enigma

Taxon classificationAnimaliaColeopteraStaphylinidae

Caterino & Vásquez-Vélez sp. n.

http://zoobank.org/90333F47-56DC-4539-866F-AA6868E7EE45

Figs 5 , 6 , 21 , 32 , 44, Map 50

##### Type material.

**Holotype male**: USA: NC: Macon Co., Jones Gap, 35.0785°N, 83.2923°W, S. Myers, vii.22.2015, sifted litter (CUAC000026531, DNA Extract MSC-2403). **Other material**: 4 males & 6 females, NC: Macon Co., 11 mi. SW Franklin, Back Country info center, VIII-17/21–1990, hardwood litter nr. dead logs, S. O’Keefe; UNHM, FMNH, CUAC. 1 female: NC: Macon Co. Highlands, vi.8.1973, Coker Rhododendron Trail, litter under rhododendron, W. Suter; FMNH.

##### Diagnosis.

This species is externally indistinguishable from *P. copelandi* except in the following male characters: metaventrite elevated anteromedially to form small but distinct median tubercle about one-fourth metaventral length behind mesocoxae (Fig. 21), metaventrite moderately flattened behind; posteroapical corner of male metatrochanter produced to form short, incurved flange (Fig. 32), the whole trochanter being somewhat parallelogram-shaped; aedeagus with sides weakly sinuate toward apex, apicodorsal ridges weakly divergent to apical corners; apical margin subtruncate to weakly emarginate; internal sac with broad band of about 18 short, sclerotized teeth. Female not definitely associated. TL 1.76–1.88mm; Max. width (EW) 0.61–0.71mm.

##### Distribution.

This species is known from the type locality, a few miles WNW of Highlands, NC, and from a second locality approximately 20 km due west.

##### Remarks.

This species is very similar to *P. copelandi*, but the male metaventral process is distinct, being located closer to the meso- than the metacoxae. The metatrochanteral processes of the two are very similar, but that of *P. enigma* is wider and situated at or distad the midpoint of the trochanter’s posterior margin. Finally the aedeagus of *P. enigma* is slightly broader and with a much squarer, only weakly emarginate apex, with a distinctly spinose internal sac, which *P. copelandi* lacks. Dissected specimens have the internal sac variably everted, so it is difficult to compare available specimens directly. However, one dissected male excluded from the type series appears to have somewhat better developed (longer) and more numerous spines on the internal sac than the type. The apex of the aedeagus of this specimen is also slightly more emarginate than that of the type. However, generalizations are impossible with such limited material. Our main basis for limiting the type series to the single male from Jones Gap is the availability of molecular data for that specimen. Interestingly, the molecular data suggest that this species may be closely related to *P. myersae*, and this is supported to some extent by the presence of internal sac armature. In external morphology, however, *P. enigma* and *P. myersae* differ greatly, given the males’ well-developed eyes and wings in *P. enigma*. Tentatively associated females have the pygidium broad and flat, the apical ventrite with strong transverse carina, concave behind, and the neck flattened beneath, but without median or lateral carinae.

One female specimen cited under ‘other material’ was initially labeled by John Wagner under his manuscript species ‘*P. suteri*’. We have not used his intended name, and cannot unequivocally associate the specimen with this species, but have left his label on the specimen.

#### Prespelea wagneri

Taxon classificationAnimaliaColeopteraStaphylinidae

Caterino & Vásquez-Vélez sp. n.

http://zoobank.org/B692F4FC-D1C8-4320-B5FC-BC954AED38A8

Figs 22 , 33 , 46, Map 50

##### Type material.

**Holotype male**: “Brasstown Bald, GA, Union Co., 11.VIII.63’, El. 2750’, B” / “Rhododendron and softwood debris” / “H.R. Steeves, Jr., J.D. Patrick Jr. collectors” / “H.R. Steeves Jr. Collection” / “PARATYPE [not]” / “P. patricki [nom. nud.]”; DNA Extract MSC-2410; deposited in FMNH. **Paratypes (13)**: 2 males, 1 female same data as type, deposited in FMNH, CUAC; 3 males and 3 females, same locality but collected September 8, 1963 from ‘forest floor debris’, deposited in FMNH; 2 males and 1 female, same locality but collected May 31, 1964 from ‘forest floor debris nr. dead wood’, deposited in FMNH; 1 female, same locality but collected October 23, 1965 from ‘forest floor debris nr rotten wood’, deposited in FMNH.

##### Diagnosis.

This species is externally indistinguishable from *P. enigma* except in the following male characters: metaventral process broader and more prominent, obviously dentate in lateral view, apically rounded in posterior view, metaventrite very weakly flattened to subconcave behind; metatrochanteral process similar to that of *P. enigma*, with a rather narrow, recurved subapical tooth. Aedeagus with sides distinctly sinuate, wide near apex, thence apically converging, apex shallowly emarginate; apicodorsal ridges not extending to distal corners; internal sac with lateral clusters of ~8 narrow spines. Female pygidium broad, very weakly convex, without median ridge or tooth; apical ventrite with very broadly and weakly bilobed transverse median carina, concave behind. Neck weakly flattened beneath, but not carinate. TL 1.72–1.92mm; Max. width (EW) 0.59–0.69mm.

##### Distribution.

This species is known from several collections on Brasstown Bald, Georgia’s highest peak (though not apparently near its peak, at a stated 2750 feet on all labels).

##### Remarks.

The distinctive internal sac armature of the aedeagus distinguishes this species from all others with fully-eyed males, and may suggest relationships to *P. myersae* and *P. minima*, which also have internal sac armature, though our phylogenetic analyses of morphological data do not unite such a group. Despite the attempted extraction of DNA from one older specimen, we have not been successful at generating a sequence for this species.

The type specimens were initially labeled by John Wagner as ‘types’ and ‘paratypes’ of his manuscript species ‘*P. patricki*’. We have not used his intended name, but have left his labels on the specimens.

#### Prespelea basalis

Taxon classificationAnimaliaColeopteraStaphylinidae

Caterino & Vásquez-Vélez sp. n.

http://zoobank.org/A9BBBBBB-C42F-47A3-A8C5-A572FD5EFB5F

Figs 20 , 32 , 45, Map 50

##### Type material.

**Holotype male**: “N.CAROLINA: Haywood Co. GSMNP, Caldwell Fork Tr. at UTM 30897 E 3940883 N. Moist forest Berlese. 3 August 2002. C.Carlton & N. Lowe” LSAM0091782, DNA Extract MSC-2418; deposited in FMNH. **Paratype male**: NC: Haywood Co., GSMNP, Cataloochee Rough Ridge Tr. [35.5927°N, 83.1374°W], July 29, 2002, C. Carlton, LSAM0092267; deposited in LSAM.

##### Diagnosis.

This species is externally indistinguishable from *P. copelandi* except in the following male characters: metaventral process indistinct; metatrochanteral process forming widened flange near basal margin of trochanter; neck convex beneath with weak median carina. Aedeagus very much like that of *P. quirsfeldi*, with sides evenly concave, apices slightly wider, and apical margin shallowly emarginate. Female not associated. TL 1.80mm; Max. width (EW) 0.67mm.

##### Distribution.

This species is known only from two localities within Great Smoky Mountains National Park. They are separated by about 5 km in the eastern part of the park.

##### Remarks.

This species is most easily distinguished by its broad and basal metatrochanteral flange (to which the species name refers). Otherwise it is extremely similar to *P. copelandi* and *P. enigma*. Its strong similarity in aedeagal shape to *P. quirsfeldi* is surprising, and may suggest the basal/default form for the genus as a whole.

This species name refers to its basally situated metatrochanteral hook.

## Conclusions

This study has revealed an unexpectedly diverse fauna of this formerly small and poorly known genus, which is still known from only a rather limited area. Further litter sampling in new areas may uncover additional species. Molecular study of relationships among known populations would greatly help delimit the species presently known, given the range of variability in many characters. We hope that future studies can obtain fresh material of *Speleobama* for inclusion in a molecular phylogenetic analysis, so that the apparent progressive reduction of several character systems may be more rigorously tested. Finally, aside from some indications of microhabitat preferences for the various species, there is nothing known of the natural history of *Prespelea*. Given their distinct sexual dimorphisms, including wing and eye development, information on their biology will be necessary to put these characters into a proper context.

## Supplementary Material

XML Treatment for Fusjugama

XML Treatment for Prespelea quirsfeldi

XML Treatment for Prespelea parki

XML Treatment for Prespelea minima

XML Treatment for Prespelea morsei

XML Treatment for Prespelea divergens

XML Treatment for Prespelea carltoni

XML Treatment for Prespelea myersae

XML Treatment for Prespelea georgiensis

XML Treatment for Prespelea copelandi

XML Treatment for Prespelea enigma

XML Treatment for Prespelea wagneri

XML Treatment for Prespelea basalis

## Supplementary materials

Supplementary material 1 Specimen data for all material examined Data type: occurrence Explanation note: This Excel file includes verbatim and interpreted (coordinate) data for all specimens of *Prespelea* examined. This dataset is made available under the Open Database License (http://opendatacommons.org/licenses/odbl/1.0/). The Open Database License (ODbL) is a license agreement intended to allow users to freely share, modify, and use this Dataset while maintaining this same freedom for others, provided that the original source and author(s) are credited.Michael S. Caterino, Laura M. Vásquez-Vélez

Supplementary material 2 Phylogenetic character data Data type: character state Explanation note: This nexus file includes morphological and molecular character data for species of Speleobamini and outgroups. This dataset is made available under the Open Database License (http://opendatacommons.org/licenses/odbl/1.0/). The Open Database License (ODbL) is a license agreement intended to allow users to freely share, modify, and use this Dataset while maintaining this same freedom for others, provided that the original source and author(s) are credited.Michael S. Caterino, Laura M. Vásquez-Vélez
