# Corrigenda: Caterino M, Vásquez-Vélez L (2017) A revision of *Prespelea* Park (Staphylinidae, Pselaphinae). ZooKeys 685: 105–130. https://doi.org/10.3897/zookeys.685.13811

**DOI:** 10.3897/zookeys.688.19867

**Published:** 2017-08-10

**Authors:** Michael S. Caterino, Laura M. Vásquez-Vélez

**Affiliations:** 1 Department of Plant & Environmental Sciences, Clemson University, Clemson, SC 29634 USA

In our recent revision of the genus *Prespelea* Park, we neglected to explicitly designate a repository for the holotype of *Prespelea enigma* Caterino & Vásquez-Vélez **sp. n.** This omission invalided its description, and that species name is not yet available. We herein rectify this problem.

## Introduction

Caterino and Vásquez-Vélez (2017) revised the Pselaphine genus *Prespelea* Park, redescribing two known species, and describing ten new ones from southern Appalachia. In one of these species descriptions we neglected to designate a type repository for the holotype of *Prespelea enigma* Caterino & Vásquez-Vélez, which, under ICZN Article 16.4.2, renders this a nomen nudum. We herein resolve this problem.

## Repositories

Specimens came from our own collections, and through loans from several institutions:

**CUAC** Clemson University Arthropod Collection, Clemson, SC

**FMNH** The Field Museum, Chicaco, IL

**UNHC** University of New Hampshire Arthropod Collection, Durham, NH

## Taxonomy

### Tribe Speleobamini Park 1951: 51

#### Genus *Prespelea* Park, 1953: 251

##### Prespelea enigma

Taxon classificationAnimaliaColeopteraStaphylinidae

Caterino & Vásquez-Vélez sp. n.

###### Type material.

**Holotype male**: USA: NC: Macon Co., Jones Gap, 35.0785°N, 83.2923°W, S. Myers, vii.22.2015, sifted litter (CUAC000026531, DNA Extract MSC-2403); deposited in FMNH. **Other material**: 4 males & 6 females, NC: Macon Co., 11 mi. SW Franklin, Back Country info center, VIII-17/21-1990, hardwood litter nr. dead logs, S. O’Keefe; UNHC, FMNH, CUAC. 1 female: NC: Macon Co. Highlands, vi.8.1973, Coker Rhododendron Trail, litter under rhododendron, W. Suter; FMNH.

###### Diagnosis.

This species is externally indistinguishable from *P. copelandi* except in the following male characters: metaventrite elevated anteromedially to form small but distinct median tubercle about one-fourth metaventral length behind mesocoxae (Fig. 21), metaventrite moderately flattened behind; posteroapical corner of male metatrochanter produced to form short, incurved flange (Fig. 32), the whole trochanter being somewhat parallelogram-shaped; aedeagus with sides weakly sinuate toward apex, apicodorsal ridges weakly divergent to apical corners; apical margin subtruncate to weakly emarginate; internal sac with broad band of about 18 short, sclerotized teeth. Female not definitely associated. TL 1.76–1.88mm; Max. width (EW) 0.61–0.71mm.

## Supplementary Material

XML Treatment for Prespelea enigma

